# Fish Immunoglobulins

**DOI:** 10.3390/biology5040045

**Published:** 2016-11-21

**Authors:** Sara Mashoof, Michael F. Criscitiello

**Affiliations:** 1 Comparative Immunogenetics Laboratory, Department of Veterinary Pathobiology, College of Veterinary Medicine and Biomedical Sciences, Texas A&M University, College Station, TX 77843, USA; smashoof@gmail.com; 2 Department of Microbial Pathogenesis and Immunology, College of Medicine, Texas A&M University, College Station, TX 77807, USA

**Keywords:** immunoglobulin, evolution, fish, teleost, immunoglobulin heavy chain, immunoglobulin light chain, shark, antibody, natural selection, humoral immunity

## Abstract

The B cell receptor and secreted antibody are at the nexus of humoral adaptive immunity. In this review, we summarize what is known of the immunoglobulin genes of jawed cartilaginous and bony fishes. We focus on what has been learned from genomic or cDNA sequence data, but where appropriate draw upon protein, immunization, affinity and structural studies. Work from major aquatic model organisms and less studied comparative species are both included to define what is the rule for an immunoglobulin isotype or taxonomic group and what exemplifies an exception.

## 1. Introduction

All jawed vertebrates share the essential fundamentals of host defense, yet there are certain distinctions amongst the components used by different groups. There are two main layers of immune responses: innate immune responses and adaptive immune responses. The innate immune system creates a fast, non-specific reaction to the pathogen infecting the host organism. If the pathogen persists despite innate defenses, then the adaptive immune system will engage the microbe with specificity and memory [1]. The adaptive (or acquired) immune system mounts to a highly discriminating, long lasting immune response to the particular pathogen orchestrated by two types of lymphocytes each driving a different arm of adaptive immunity. T cells are major components of cell-mediated immunity whereas B cells produce immunoglobulins (Ig) that are key elements of humoral immune responses [2]. T cell receptors and B cell receptors (plasma membrane anchored Ig, as opposed to secreted Ig that is also known as antibody) are extremely diverse molecules at their antigen binding tips. This vast structural diversity is the result of their membrane-distal variable domains being the product of somatic recombination of variable (V), diversity (D) and joining (J) gene segments [3,4].

The immunoglobulin superfamily-based adaptive immune system evolved in the ancestors mammals share with cartilaginous fish (such as sharks) nearly one half billion years ago. Cartilaginous fish, bony fish, and tetrapods employ this system characterized by Ig, T cell receptors (TCR), cytokines, and major histocompatibility complex class (MHC) I and II molecules [5,6]. The spleen is the consistent peripheral lymphoid organ throughout jawed vertebrates, and while T lymphopoeisis is unfailingly thymic, B cells mature in a myriad of organs, including shark epigonal, skate Leydig, tetrapod bone marrow, bird bursa of Fabricius, sheep ileal Peyer’s patches, rabbit appendix and teleost head kidney [7].

In this review, we will summarize some of the important structural, functional and genetic features of fish immunoglobulin classes. We then briefly will address the mechanisms of Ig repertoire diversification in some fish model species.

## 2. Immunoglobulins

Immunoglobulins or antibodies, which play a vital role in adaptive immune responses, are heterodimeric glycoproteins belonging to the broad Ig superfamily (IgSF) [8,9]. Antibodies were first reported by von Behring and Kitasato in 1890 as an agent in the serum that could neutralize diphtheria toxin [10].

The basic structure of Ig consists of two identical heavy (H) chains and two identical light (L) chains (although we will discuss exceptions in shark and camelids). Both heavy and light chains contain one N-terminal variable domains (V_H_ or V_L_) and one or more C-terminal constant domains that form the constant region (C_H_ or C_L_), all domains adopting the hallmark fold of the IgSF. Variable domains participate in antigen recognition and the domains that comprise the constant regions mediate effector functions of the antibody molecule. Such humoral immunity mediated by Ig includes opsonization of pathogens for destruction by phagocytes, neutralization of toxins and viruses, and activation of the complement cascade. The variable region paratope (that engages the epitope of the antigen) is formed by variable domains of IgH and IgL (one variable domain in each chain). Constant regions consist of constant domains of both IgH and IgL as well. While IgL have one C_L_ domain, the number of constant domains of IgH varies among different immunoglobulin classes [11].

There are three hyper variable regions within each V_L_ and V_H_ domains termed complementarity-determining regions (CDR). The three CDR segments of V_L_ domain and the three CDR of V_H_ domain together contribute the majority of the antigen binding site of the Ig molecule [12]. Different combinations of CDR in V_L_ and CDR in V_H_ diversify the antigen binding site amongst the millions of different antibodies in an individual. Antibody repertoires are tremendously diverse which enables them to recognize a vast array of distinct antigens. We will discuss the mechanisms responsible for creating the huge variability CDR sequences and thus of antibody repertoires.

Inter-chain disulfide bonds between cysteines hold together IgH and IgL and also the two IgH. The number and location of these bonds differs amongst Ig class and amongst species. There are also intra-domain disulfide bonds within each domain of each chain [13]. The assembled chains of antibody assume a “Y” shaped quaternary structure in which amino terminal fragment antigen-binding (Fab) and fragment crystallizable (Fc) stabilize using a flexible polypeptide hinge region between the first and second CH domains. The flexibility of the hinge region will allow the Fab fragments to pivot freely and independently from one another, allowing the binding of two identical epitopes [14]. This flexible bivalency of the Ig facilitates crosslinking of multivalent surface epitopes of pathogens.

As alluded to earlier, the C_H_ are engaged in effector functions of antibody. Antibodies are categorized into specific classes or isotypes based on the structure of their C_H_ regions. Different classes of antibodies conduct different effector functions as different C_H_ allow different binding to Fc receptors of specific types of immune cells, complement proteins, and molecules facilitating transport across mucosal surfaces, into the colostrum and into fetal circulation.

In mammals, five isotypes of Ig have been described and are named IgM, IgD, IgG, IgA, and IgE. Each perform distinct effector functions at particular anatomic locations. For instance, IgM and IgD are naïve B cell surface antigen receptors and IgM primarily activates complement and is chiefly found in the blood. IgG is the most predominant isotype in the blood and extracellular fluid. IgG also activates the complement system and opsonizes pathogens for phagocytes. Additionally, it mediates antibody dependent cell cytotoxicity by natural killer cells. IgA plays a major role in mucosal immunity and is the principal isotype secreted into the gastrointestinal and respiratory tracts. IgA essentially functions as a neutralizing antibody at mucosal surfaces. Unlike IgG, very little IgE is present in blood or extracellular fluids, but IgE is relatively small and can easily diffuse out of blood and penetrate into tissues. There IgE principally defends against helminthic parasites and mediates immediate hypersensitivity by coating mast cells [12,15]. Mammalian IgL have two isotypes: κ (kappa) and λ (lambda), where little if any functional distinction has been ascribed.

In lower vertebrates, a smaller number of antibody classes have been reported. In cartilaginous fish, three heavy chain isotypes have been detected: IgM, IgD/W, and IgNAR [16]. Thus far, in teleost fish, three different Ig heavy chain isotypes have also been identified: IgM, IgD, and IgT/Z. Not every class is expressed in each fish species studied [17]. Sarcopterygian fish display IgM and/or IgW, and variants thereof. The Ig classes of fish will be discussed in more detail in the following sections of this article. The lymphocytes of jawless agnathans (lamprey and hagfish) employ antibodies that are genetically and structurally not orthologous to those of jawed vertebrates (gnathostomes). These variable lymphocyte receptors (VLR) contain leucine-rich repeat domains instead of the IgSF and are structurally closer to toll-like receptors than gnathastome Ig [18,19,20]. Therefore, we will not cover the VLR here except to acknowledge this emerging area of comparative immunology and immunotherapeutic development [21,22].

## 3. Cartilaginous Fish IgH

Cartilaginous fish are phylogenetically the most ancient vertebrates capable of undergoing an IgSF-based antibody response. The cartilaginous fishes (Chondrichthyes) diverged from a common ancestor with the lineage leading to other bony vertebrates ~450 million years ago (MYA) [23]. They are the most distantly related group to mammals having an immune system grounded upon Ig [24], TCR, the MHC, as well as recombination activating gene (RAG)-mediated rearrangement, somatic hypermutation (SHM), and the presence of primary and secondary lymphoid tissues [25]. Cartilaginous fishes are divided into two subclasses, the Holocephali (ratfishes, chimaeras, elephant sharks and rabbitfishes) and the Elasmobranchii (sharks, skates and rays) [26]. Cartilaginous fish do not have bone marrow but instead it uses the epigonal organ associated with the gonads and the Leydig organ embedded within the walls of the esophagus as the tissue responsible for B cell lymphopoiesis [27,28]. In the absense of lymph nodes, the white pulp of spleen is the major secondary (or peripheral) lymphoid tissue in the cartilaginous fish, but the gut associated lymphoid tissues have also been implicated [7].

Thus far, three heavy chain isotypes, IgM (µ), IgW (ω; orthologous to IgD), and the cartilaginous fish lineage-specific isotype IgNAR, have been identified in Chondrichthyes [25,29].

### 3.1. IgM

A potential candidate for IgM was discovered 50 years ago in the spiny dogfish shark (*Squalus acanthias*) serum [30]. IgM is considered the most ancient antibody molecule which shares similar functions in all gnathostomes [31]. IgM was thought to be used in all jawed vertebrates, until work in the coelacanth found it to be extinct representative of the Sarcopterygian lineage as the only known jawed vertebrates to lack IgM [32]. Both during ontological development and in the course of adaptive humoral immunity, IgM was the first isotype expressed, and it is the major B cell receptor on the surface of both conventional B2 and “innate” B1 and marginal zone B cells. IgM is known for conserved form and function across all vertebrate classes. However, it does display different polymerization states in secreted antibody and a significant splice form in bony fish. In cartilaginous fish, IgM is a major serum protein that accounts for more than 50% of serum protein [24].

**Figure 1:**
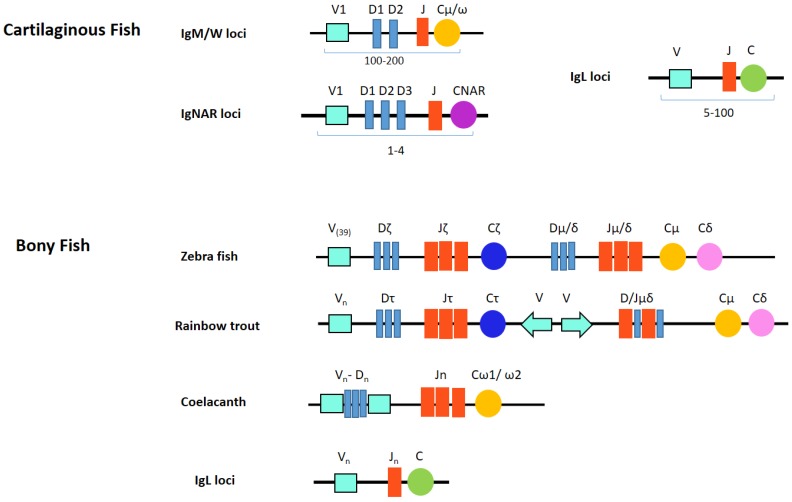
Both multiple cluster and translocon organizations are used in fish Ig loci.

Chondrichthyan IgM loci adopt a multiple mini-cluster organization instead of the single translocon locus of bony vertebrate classes [33]. Most species appear to highly diversify IgM and can have tens of germline genes, possibly up to 200 [25]. Gene numbers differ between individual species and somewhat between individual animals within the species, but five functional IgM subclasses always seem to be present. Each cluster consists of just a few gene segments (V_H_-D1-D2-J_H_) and one C gene (Figure 1). Although most miniloci appeared individually capable of rearrangement, many other clusters are partially (VD-J, VD-DJ) or fully (VDJ) recombined in the germline. For example, there are no canonical germline-joined IgM in nurse shark. Their IgM H chain repertoire is based on junctional diversity at 15 loci from five families and, successively, somatic hypermutation [34].

IgM1_gj_ is a subclass of IgM reported in nurse sharks that is expressed in high amounts as a monomer in the sera of neonates and has only three C domains [28,33]. This germline-rearranged gene encodes both monomeric and dimeric serum antibodies [26,33]. This form of IgM is consistently expressed in the epigonal organ but its levels decline in the spleen as the animal ages [35]. Unlike canonical IgM, the C_H_2 domain is missing in IgM1_gj_ from IgM, similar to the loss of the domain from mammalian IgG. The V from the IgM1_gj_ cluster is completely joined in the germline at the D-J and V-D juxtapositions [25].

Sharks have IgM in two different states in the serum, termed 7S and 19S, due to their different sedimentation coefficients. The monomeric 7S and the pentameric 19S are present in approximately equal amounts despite independent production from the same set of IgH loci [24]. Sharks appear to be able to orchestrate a switch from secreted 19S pentameric IgM to secreted 7S monomeric IgM in the course of a humoral response [36]. This IgM response of sharks was originally thought to be very different from mammals, particularly because the kinetics of the climb in titer was slower to rise than in endotherms. Serum IgM levels climbed slowly, taking months for an antigen-specific titer to plateau [37]. Thus, cartilaginous fishes appear singular amongst vertebrate classes in their significant production of both monomeric and polymeric forms of the same heavy chain isotype [38] (although bony fish do secrete some IgM monomers [39]).

In the nurse shark, Ginglymostoma cirratum as in mammals, the J chain is present only in pentameric IgM although unlike mammalian J chain it may not be involved in IgM secretion [40]. The shark Tm and Sec forms of IgM have the same number of IgSF domains [25]. Typically, the heavy chain of both cell-bound and secreted form of this protein is composed of an Ig-superfamily domain corresponding to the V region and four domains of the C region [41]. Most studies showed an antigen-specific increase in both forms of IgM, as well as an increase in the relative ratio of monomeric to pentameric IgM during the immunization course [36]. The IgM expression increases over the immunization course; therefore, the IgM level in adults are much higher than neonatal sharks.

IgM is usually expressed in eye, gills, intestine, liver, pancreas, peripheral blood leukocytes, spleen and testis but not in brain, heart, kidney, muscle, skin and stomach [41]. IgM has been considered to have important roles in more ancestral clades of vertebrates in addition to antigen binding. One hypothesis is that IgM has a role in blood osmoregulation analogous to the physiology of albumin in other vertebrates [42]. Shark IgM antibodies have been shown to be active in lytic, opsonic and antibody induced cytotoxicity-like reactions. This killing is observed through phagocytosis which is mediated by both 7S and 19S IgM antibodies. Throughout evolution, IgM reacts with particulate antigen and through binding FCµ receptors on the surface of leukocytes enhancing phagocytosis. Neutrophils appear most frequently associated with IgM mediated phagocytosis [43,44].

### 3.2. IgW

IgW, which was first discovered in skates [45,46,47], later in shark [48,49], and then coelacanth [50,51], has gone by many names (IgX, IgNARC or IgR) before being proposed as an ortholog of IgD [29]. Nearly 500 MYA in the first jawed vertebrates IgM and IgW evolved in a relatively short period of time [32]. IgD is orthologous to IgW, found only in cartilaginous fish and lungfish, demonstrating that IgD/W, like IgM, was likely present in the ancestor of all living jawed vertebrates [29]. IgW has been detected to be highly expressed in the shark pancreas, much less in muscle, and faintly in gills, kidney, spleen and testis [41]. Cartilaginous fish IgW exists as either a seven-domain or three-domain form [45,49,52], but in lungfish it can have two to at least 11 C domains [53]. Similar to IgM, IgW is encoded at loci employing the multiple cluster organization. In nurse shark, eight loci encoding the IgW C_H_ have been characterized. Each cluster contains six to eight C domain exons in addition to V, D and J segments. The IgW H chain long form is composed of seven domains in all cartilaginous fish so far studied [54] and eight domains in the lungfish [55] which is homologous to IgW-long. An IgW short form consisted of three domains which appears to be spliced differently than the long form [46,47]. Two cell bound forms (containing two and four Cδ) and at least seven secreted forms (containing two, four, six or eight Cδ as well as a six Cδ form that lacks a V region) have been found thus far, all of which can be generated by alternative RNA splicing [26].

The V-less IgW form (IgWΔV), with a leader sequence spliced directly to Cδ1, has been described in both the spiny dogfish and the nurse shark. In spiny dogfish, these V-less transcripts represented 8% of the IgW transcripts analyzed, while the V-less transcript levels in nurse shark could only be detected using RT-PCR. In this regard, the functional significance of the different IgW forms can only be determined once IgW-specific reagents, such as mAbs, become available [56].

Interestingly, IgW V is expressed in both the thymus and the spleen of sandbar shark [48], possibly explained by Ig-TCR chimeric transcripts described with IgW V and TCRδJ-C [57]. IgWshort is most highly expressed in the spleen. Also in unimmunized adults, IgWshort transmembrane (Tm) and secreted (Sec) forms are expressed in the pancreas which suggests it could have a role in gut mucosal immunity [54]. The short (two Cδ) secreted form has a long, cysteine-rich tail that is very different from the Sec tail of other Igs (IgD/W or any other known isotype) suggesting its effector function is different from the other isoforms [26]. In situ hybridization demonstrated that Igω is co-expressed with J-chain in the adult epigonal tissue, suggesting that IgW may be expressed as a multimer [56].

### 3.3. IgNAR

IgNAR (new or nurse shark antigen receptor) was first reported in 1995 by the Flajnik group in nurse shark and contains one V and five C domains. The carboxy-terminal C domains have homology to those of IgW long [25]. Most notably, it is found as a dimer in serum without light chains [58]. IgNAR has been found in intestine, liver, pancreas, peripheral blood leukocytes, spleen and testis [41]. Similarly as in IgM and IgW, IgNAR genes are organized in the multiple cluster format (Figure 1). Each genomic IgNAR cluster comprises one V segment, up to three D segments and one J segment and one set of C segments. Rearrangement occurs solely within this cluster resulting in a V(D1D2D3)JC assembly. Hence, up to four RAG-mediated rearrangements can proceed to generate the mature exon encoding the complete V NAR domain [59,60,61]. Consequently, diversity in both sequence and length of the primary repertoire is focused in CDR3. Extensive junctional diversification through N-region addition, P-nucleotide addition, exonuclease trimming, and D-region rearrangement further expands the heterogeneity of the primary repertoire of IgNAR [59]. The multiple rearrangement events vary CDR3 length greatly (range 5–34 aa). IgNAR V elements then undergo much somatic hypermutation in response to antigen [62].

IgNAR V domains of 14 kDa contain the entire antigen binding paratope. The single small domain facillitates recombinant protein expression and lends stability to the molecule, both advantages over the canonical IgHV-IgLV binding surface of most antibodies [63]. The most unique feature of V-NAR paratope is the absence of a CDR2 loop and of the two β strands, C′and C″, associated with it. Instead, a “belt” span is formed around the middle of the β-sandwich IgSF structure [64]. Due to this deletion in the framework of the 2-CDR2-region, the β-sandwich fold only consists of eight instead of 10 β-strands, which made the IgNAR V domain the smallest antigen binding unit known at the time [65]. (since ultralong-CDR3 cattle antibodies have claimed this title [66,67]).

The V domains of IgNAR are classified into three main categories, called isotypes I, II and III, based on the disulfide bond pattern and the positioning of non-canonical cystein residues. Diversity is concentrated in the long CDR3 at the expense of variety in CDR1 and CDR2 in all three types of IgNAR. This CDR3 ranges in length from five to 23 amino acids, although 15–17 residues are more common. Thus, shark IgNAR CDR3 lengths are similar to those of camelid V_H_H in being much longer than most vertebrate IgH CDR3 [68] (save bovine ultralong CDR3 [67]).

NAR V is unique in that it has an exceptionally small CDR2 and poor conservation of those residues responsible for V_H_/V_L_ and V α/β dimerization in typical Ig and TCR V, respectively. NAR also displays a uniquely flexible constant (C) region [60] but demonstrates high biophysical stability, solubility, and ability to bind to a variety of antigens including recessed epitopes that are non-accessible by traditional two-V domain paratopes [64]. The unusually high stability of IgNAR could be adapted to the high urea concentration in elasmobranch tissue [69].

Two transmembrane forms of IgNAR have been identified, with either three or five C domains [41,70]. There is much less IgNAR than IgM in serum (0.1–1 mg/mL) [26].

IgNAR consists of H chain dimers or multimers [52]. Being without an L chain, each chain of IgNAR carries a single-domain V region that each bind to antigen independently [71]. It is not yet known if IgNAR multimers require J chain for their formation [26,72]. It has been shown that in some species of shark a high affinity antigen specific IgNAR response can be elicited in response to antigenic challenge [73,74]. The shark IgNAR platform can recognize epitopes that the immune systems of mammals cannot, thereby offering opportunities to isolate novel binders and novel cryptic epitopes, potentially with high affinities, that could not otherwise be produced. This characteristic of IgNAR make it a good candidate for therapeutic applications, particularly for viruses where broadly neutralizing humoral immunity is desired but difficult to achieve [75].

## 4. Bony Fish IgH

Almost every aquatic niche on the planet has been colonized by Osteichthyes, or bony fish. Over 40,000 species exist and account for more than 50% of all known vertebrate species [23]. The infraclass of Teleostei represents approximately 96% of the species of this superclass. Osteichthyes are further split into the Sarcopterygii or “lobe-finned” fishes (which include the coelacanths and lungfishes which are closest in relation to tetrapods), and the Actinopterygii or “ray-finned” fish, the latter where most fish are placed [26,76].

Like chondrichthyes, Osteichthyes have thymus and spleen. Osteichthyes employ the head kidney or pronephros for hematopoesis and B lymphopoiesis [7].

Fish B cells express TM BCR on their plasma membrane and in response to antigen will secrete antigen-specific Sec antibodies [77]. Three classes of Ig have been identified in teleost fish. These are IgM, IgD, and IgT/Z (for Teleost/Zebrafish), which is specific to fish [78]. IgM and IgD are ubiquitously found in most fish species as well as most vertebrate classes, leading to hypotheses that they are ancestral isotypes. The fundamental immune molecules, including Ig, TCR, MHC, AID and RAG, appear similar in cartilaginous fish, bony fish and mammals [1].

Ig of teleost fish is found in the skin, gut, gill mucus, bile, and systemically in the plasma [79,80]. Most fish respond to primary antigenic challenges by producing specific antibodies, whereas the response to secondary challenge is generally faster and of greater magnitude. This makes vaccination an attractive strategy for control of infectious disease in the aquaculture industry [81].

### 4.1. IgM

The IgM class of antibody has long been considered the most ancient and is the most prevalent Ig in fish plasma [31,78]. However, the complexity and large size of IgM has led to some doubts that it was the primordial Ig [82]. IgM was the first Ig class identified in fish. It can be expressed on the surface of B cells or Sec as antibody. IgM in either serum or mucus is multimerized into a tetrameric form. The Tm form of IgM is one domain shorter than the secreted form due to alternative splicing and does not have the Cµ4 domain. Interestingly, this does not seem to affect the effector function of this isotype [83]. Some early studies report IgM monomers in the grouper, *Epinephelus itaira* [84] sheepshead, *Archosargus probatocephalus*, and trout, *Oncorhynchus mykiss* [85,86,87].

Teleost serum contains natural IgM antibody before immunization, but antigen exposure with T cell helps drive release of specific tetrameric IgM [88]. Serum IgM concentrations have been found between 800 and 9000 µg/mL in teleosts [89]. Bony fish IgM is secreted in redox forms that may affect binding affinity [90]. Antibodies are also found in the channel catfish epithelial mucus, and specific antibody was detectable in higher titers in the intestinal extract from orally immunized fish, than in the sera and vice versa, parenteral immunization resulted in high and persistent antibody titers in the serum, but not in the intestinal and cutaneous secretions [91].

IgM contributes to both innate and adaptive immunity in fish. IgM effector functions include complement activation which both lyses and opsonizes pathogens [92,93]. IgM also mediates agglutination for phagocytosis and removing pathogen, and cellular cytotoxicity [89]. IgM responses in serum and the intestine can be bolstered by dietary addition of arginine and glutamine at least in the channel catfish [94].

Teleost IgM lacks the J chain and thus tetrameric IgM is polymerized by interchain disulfide bonds [90]. It is thought that the number of these disulfide linkages may be determined by the affinity of the BCR for specific antigen, where higher affinity tracks with more cysteine linkages. The level of effector function of the molecule (which can be inducing cytotoxicity, complement activation, and phagocytosis through effector cells) may be affected by the stability afforded by these inter-IgM covalent bonds [89,95]. Following immunization, an increase in IgM serum titer and weak improvement in binding affinity occurs and an anamnestic response had been detected [89,96,97,98]. The magnitude of affinity maturation is typically much less than that obtainable in a mammalian response. Two sub-isotypes of IgM (salmon CHA and CHB) in Atlantic salmon. One possible explanation for these two closely related salmon CH genes is the tetraploid genome state of salmonids [99].

### 4.2. IgD

IgD was first discovered in human serum in 1965 [100,101], and we know only a little more of its physiology now than then. One proposed role for Sec IgD is high affinity binding to an as yet to be identified receptor of basophils and induce the production of antimicrobial, opsonizing, pro-inflammatory, B cell activating factors [102]. When initially described, IgD was thought to be a recently evolved isotype of some mammals. However, in the last decade, IgD has been found in many vertebrate classes suggesting that it may be as old as IgM [29].

**Figure 2:**
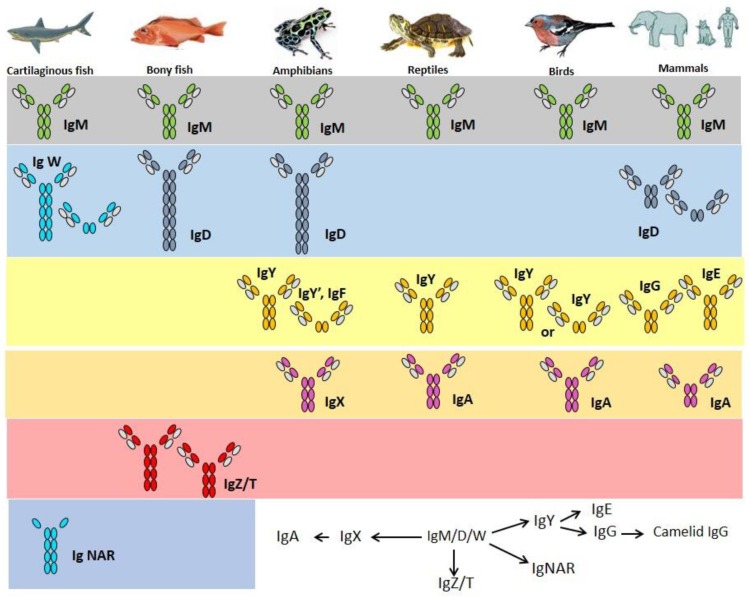
Immunoglobulin heavy chain isotypes of fish and their proposed [110] relationships with the isotypes of other vertebrate groups, which is still quite controversial.

If IgW is indeed orthologous to IgD, the isotype may just have been lost in birds [103] (for example duck and ostrich [104,105]) and in few mammalian species (e.g., elephant and opossum [106,107], overview in Figure 2). Bony fish contain many Tm and Sec IgD variants [108,109]. Channel catfish was the first teleost in which IgD was identified [82]. The concentration of IgD in serum is 2–80 µg/mL. IgD secreting cells can be found in anterior and posterior kidneys, spleen and gills [56].

A unique feature of teleost IgD is that it is a hybrid of the Cµ1 domain followed by a different number of Cδ domains depending on the species of fish. In fact, the fish IgH δ locus contains a rearranged VDJ spliced to Cμ1, a variable number of C domain encoding exons (depending upon the species), and a Tm tail [111,112,113,114,115,116]. The IgD heavy chain has not been found yet in fish without Cµ1 [77]. The number of fish IgD Cδ can range from 7 to 17 (in zebrafish). IgD does not contain a hinge. It was thought that teleost IgD is expressed only in Tm form until both secreted and membrane IgD was discovered in the channel catfish and subsequently secreted IgD was quantified from rainbow trout [109,111].

The genomic organization of teleosts δ exists in a broad variety. For example, in Atlantic cod, there was a tandem duplication of a portion of the δ locus including the δC1 and δC2 domains [113]. In channel catfish, the δ locus contains two copies of delta genes, highly similar in sequence and each containing a tandem duplication of δC2-δC3-δC4. One of these two loci encodes the Tm form of δ and the other one possesses an exon for Sec δ [111]. In contrast, the stickleback IgD locus has seven C domain exons and none is duplicated [117].

### 4.3. IgT/Z

Bony fish are the only animals that produce IgT/Z. IgT/Z was first identified in rainbow trout (IgT) and zebrafish (IgZ) [118,119]. Subsequently, the isotype was discovered in most model species of teleost, with the notable exceptions of medaka and channel catfish. The IgT/Z organization within the fish IgH locus encodes a varying number of C domains in different species. For example, fugu has two C domains [120], while the common carp IgZ2 subclass is a chimera of IgM and IgZ with two constant regions yet the first constant region is Cµ1 of carp and the second constant region is very much similar to zebrafish Cζ4 [121]. The carp IgZ2 chimera seems more abundant in mucosal sites whereas IgZ1 is found in systemic organs [122]. The stickleback IgT/Z encodes three C domains [117]. However in most species characterized to date IgT/Z has four C domains, as in the originally described rainbow trout and zebrafish [123].

Several datasets support the idea that IgT/Z is the most important immunoglobulin of mucosal surfaces in bony fishes if not a dedicated mucosal immunoglobulin isotype [124]. Studies in trout showed that in animals surviving an intestinal parasite infection large number of IgT^+^ B cells were detected, while the number of IgM^+^ B cells did not change. Also, the number of IgT (but not IgM) in the gut of surviving animals was increased. In contrast, the titration of parasite-specific IgM was very high in the sera but not the same for IgT in the sera. Lastly, IgT was found on the surface of the gut bacteria [125]. IgT, a monomer in trout serum but a tetramer in mucous, is able to pass through the mucosal epithelium using a fish polymeric immunoglobulin receptor (pIgR) [125].

Recently, we identified IgT in *Thunnus orientalis* (Pacific bluefin tuna). Tuna IgM and IgT contain four C domains each. There are four V_H_ gene segment families, and three V families were used by both IgM and IgT but IgM exclusively rearranged to one family. IgT and IgM typically share V segments with each other but not D and J segments. Notably, the same D segment appears to be used by both IgM and IgT rearrangements in tuna, unlike other groups of fish studied [124]. IgT^+^ B cells extensively populate teleost SALT (skin associated lymphoid tissue) and are responsible for secreting polymeric IgT into the skin mucus to protect the skin from infectious microbes [126]. Thus, teleost IgT represents the phylogenetically oldest known immunoglobulin isotype that is specialized for gut mucosal responses [6].

## 5. Fish IgL

### 5.1. Cartilaginous Fish IgL

There are two identical IgL chains in a canonical antibody molecule. Similarly to IgH gene organization in cartilaginous fish, IgL chain genes are arranged into multiple clusters containing one V, one J and one C segments [127]. Four light chain isotypes named kappa κ (previously called NS4 or type III), lambda λ (NS3 or type II), sigma σ and sigma-2 (σ-2 NS5, type I or σ-cart) have been identified in cartilaginous fishes. IgL chain consistently is comprised of one V and only one C domain. A covalent disulfide bond between cysteines links the C_L_ and the IgH C1, and the antigen binding domains of V_H_ and V_L_ associate non-covalently [128].

In cartilaginous fishes, some IgL loci are capable of rearrangement while others contain germline fused V-J genes [129]. In all species studied, λ (type II) L chain genes are all germline-joined, but there are widely varying numbers of IgL genes in different species for different isotypes. The σ-2 (type I) genes are all joined in skates and split in the horn shark. Nurse shark λ genes are completely joined, but there are only six loci. IgLκ (type III) genes are expressed at the highest levels, and it is estimated that there are approximately 60 genes present in the genome. Most of the nurse shark κ genes are split, but a few are germline-joined [25,130]. There are only three expressed σ-2 (type I) genes, two being split, each consisting of a V and J gene segment that can recombine and one C region, and one germline-joined V-J in-frame and the fourth locus is a pseudogene in this species [131].

Sterile transcripts of non-rearranged IgH and IgL are more easily detected from neonatal tissues, betraying early locus activation and accessibility. Promotors only appear to be associated with the V gene segments [71]. The IgL chains are found at different concentrations in different species. The κ (type III) genes are expressed at highest levels in nurse sharks [130] σ-2 (type I) in horn sharks, and λ (type II) in sandbar sharks [25,132]. The nurse shark σ-2 (type I) and at least one of the κ (type III) chain germline-joined genes are expressed early in development, and this σ-2 (type I) germline-joined light chain is the preferred heterodimerization partner of the IgM1gj IgH which is expressed in neonates [25].

Similar to IgH, functional V_L_ exons are made by stochastic combinations of V and J gene segments at the level of the somatic DNA in the developing lymphocyte. The gene segments are flanked RSS, recognized by the RAG enzymes, which initiate and play a critical role in the gene rearrangement. Affinity maturation via SHM can further diversify V_L_ as well as V_H_ after antigen exposure. Similar to the IgH V hypervariable, CDR loops dictate the contribution to antigen specificity of the IgL V. CDR1 and CDR2 are encoded solely by the V gene, but CDR3 is programmed by the V-J rearrangement junction and therefore it is the most varied CDR for IgL as well [128].

The most common of the diversifications at the V-J juncture in shark IgL are N (nontemplate encoded) additions which are catalyzed by terminal deoxynucleotidyl transferase (TdT) at the IgH V(D)J junctures as well. Artemis and the DNA-dependent protein kinase resolve the DNA hairpins at the ends of V(D)J coding segments randomly, which donate P (palindromic) nucleotides. Finally, the exonuclease activity of several DNA repair enzymes removes nucleotides, even while TdT is adding them. Thus, several mechanisms contribute to CDR3 diversity in the IgL protein with immunogenetics at the V-J recombination site [133].

Studies on the clearnose skate *Raja eglanteria* showed that developmentally, IgLσ-cart showed moderate expression throughout the first 11 weeks of embryonic life with a peak at week eight, while IgLλ was nearly absent save for a peak at week eight. Differential expression of IgLσ-cart and λ were seen in individual tissues in this skate as well, IgLλ being more abundant in Leydig organ whereas IgLσ-cart is higher in the gonad and liver; both isotypes being high in spleen. In all tissues, IgLσ-cart expression dominated IgLλ at the eight-week embryo and hatchling stages, a pattern reversed in the adult skate [134].

### 5.2. Teleost Fish IgL

Among teleost species, the humoral immune system of salmon, medaka, trout, zebrafish, catfish and cod have been best characterized. To date, four IgL isotypes, orthologous to λ, κ (previously called L1, L3, F or G, and now called Ig κF and Ig κG) σ (otherwise known as L2) and σ-2, have been identified in bony fish. When searched for, κ and σ have been found in most bony fish. Igλ, on the other hand, seems to have been lost from most teleost lineages (cod and catfish are two notable exceptions). The σ-2 isotype (formerly known as σ-cart) was recently identified in the coelacanth. As no λ5 or VpreB surrogate light chain components have been found in fish, it remains to be seen how ordered chain rearrangement and testing is conducted and allelic exclusion maintained [26,51].

Bony fish IgL chain genes can occur in the translocon organization or the multiple cluster organization [128,135]. As in cartilaginous fish, teleost IgL genes are arranged in (V-J-C) genomic clusters, although there are distinct differences. Bony fish IgH are in a translocon organization and but their IgL can be multiclustered. Additionally, V_L_ are often in an inverted orientation to JL, teleost IgL could use inversional V-J rearrangement within or between clusters [136]. Teleost IgL genomic organization with closely linked clusters of (V-J-C) offers several recombination possibilities for receptor editing and locus silencing [137].

At least some Igκ V elements are in opposite transcription orientation with respect to their J and C genes, again implying that this reverse orientation allows teleost Igκ genes to rearrange via inversion rather than deletion. Also, the numbers of clusters vary in the different species and between the different IgL isotypes. Most teleost IgLκ gene clusters, as represented by the catfish, contain one or two V elements, a single J gene, and a single C gene [56].

CDR1 of IgLσ V is not as long as CDR1 in other isotypes in most vertebrates (save chicken V_L_ κ) and CDR2 of σ is longer. These two germline-V encoded CDR of IgL isotypes show length conservation throughout vertebrates. While Vσ has a longer CDR2 but shorter CDR1, these CDR lengths are flipped for Vλ and Vκ. It was therefore hypothesized that the different L chain isotypes evolved independently to provide different structures when paired with V from IgH [128].

Recently, Zhang et al. showed in rainbow trout that there are eight IgL sub-isotypes (IgLκG1, IgLκG2, IgLκG3, IgLκF1, IgLκF2, IgLs1, IgLs2, IgLl), belonging to four IgL isotypes (IgLκG, IgLκF, IgLs, and IgLl), respectively. The V_L_ domain of trout IgLσ1 is longer than other isotypes. They have also shown that there is a preferential combination of the IgL and IgH chains within trout IgM and IgT. In the head kidney, within IgM^+^ cells, IgLkG2 was the dominant IgL subisotype, while IgLκG1, IgLκF2, IgLσ1 and Igλ showed equivalent transcriptional levels. In the IgT^+^ cells, even though IgLκG3, IgLκF2, IgLσ1 and Igλ showed similar transcriptional levels with those in the IgM^+^ cells, the expression level of IgLkG2 was five-fold less. A similar result was observed in trout peripheral blood leukocyte. Catfish serum IgM is associated with IgLκF and IgLκG at a ratio of 60:40 [135].

## 6. Fish Ig Locus Organization

The rapid diversification (or “big bang”) of the vertebrate immune system is hypothesized to have occurred <500 million years ago, with the incorporation of a transposon containing recombinase activating genes into a primitive Ig coding sequence [138]. Gene duplication and evolution of the immune effector molecules rapidly could have followed, along with recruitment of other proteins to maximize antibody diversity, and the addition of increasingly sophisticated levels of control and complexity [139]. The discovery of RAG genes in echinoderms [140] moves at least some events in the genesis of the system to an earlier point in deuterostome evolution, unless the RAG transposon integration occurred more than once [141].

### 6.1. Cartilaginous Fish Ig Locus Organization

The Chondrichthyes contain the most IgH and IgL multi-clusters, and as many as two hundred (V-(D)-J-C) clusters have been predicted to exist in the genome of some species [136]. The immunoglobulin genes of all elasmobranchs studied are arranged in the cluster configuration, rather than the translocon organization typical of the mouse or human. Each cluster contains one variable V segment, a number of diversity (D) segments, one J segment and one set of constant regions required to generate that particular Ig chain [142]. All available data suggests RAG mediated VDJ/VJ rearrangement occurs exclusively within a cluster and not between clusters [62,143] but isotype switching can occur between clusters [144]. V, D and J elements in shark and ray Ig clusters tend to be in the same transcriptional orientation. This necessitates deletional (rather than inversional) rearrangements, which could restrict such rearrangements to single clusters. The situation is similar for the L chain genes (except they lack D segments). In some instances, the segmental elements within a cluster may be fully (VDJ or VJ) or partially (VD-J) joined in the germline. Joined genes are derived from canonical “open” Ig genes that RAG acted upon in germ cells [145], and appears to be peculiar to the Ig genes of cartilaginous fish [25].

As discussed, the numbers of clusters can vary considerably among species. For instance, compared with horned shark, there are only 9–12 distinct, functional Igµ and three pseudogenes in nurse shark [71]. Whereas within a cluster, recombining gene segments such as V_H_-D1, V_L_-J_L_, or VNAR-D1 are 250–500 base pairs apart, the clusters themselves are spatially distant, that is, 120 to >200 kilobases between Igµ, and 82 to >200 kilobases between Igµω [146]. Clusters can be spread amongst many chromosomes [147].

In the nurse shark, there are about 15 IgM H chain loci per genome, and every functional gene contains one V_H_, two D, and one JH gene segments located within ~2 kilobases. Data from this species suggest that allelic exclusion at IgH loci must be regulated differently than in mammals as ordered long distance recombination and chromatin contraction appear to be absent in sharks. Allelic exclusion appears to still occur in shark B cells though, where only one or a few of its many IgH loci rearrange in any one cell [148].

### 6.2. Bony Fish Ig Locus Organization

In Osteichthyes, IgH chain genes are found in a translocon organization. An array of V elements are 5’ of possible D segments, followed by J segments and exons encoding constant domains for one isotype. V(D)J rearrangement proceeds in the primary lymphoid tissues to generate combinatorial diversity [3].

In catfish, unlike the teleost Igκ genes, each of Igσ and Igλ are encoded by only two clusters. Similarly, Atlantic cod Igλ genes are predicted to be encoded by two or three clusters. In catfish IgM/IgD^+^ cells were found only to express Igσ. However, catfish, Atlantic cod, and rainbow trout are the only teleost where Igλ genes have thus far been identified [149]. In zebrafish IgLλ is absent and IgL (V_L_-J_L_-C_L_) clusters span five separate chromosomes (#1, #12, #19, #24, and #25), and IgL V is in the same or opposite transcriptional orientation as J and C [150]. Zebrafish IgLκ gene clusters are longer due to multiple V_L_ gene duplications. All together there are twelve IgκL1 genes scattered over three different chromosomes, and the different Igκ clusters can contain from one to four V elements. The zebrafish IgLκ L3 genes are organized slightly differently: in two clusters of (V4-J-C-V2) and one (V2-J-C-V2), i.e., there are 10 V elements. In contrast, the zebrafish Igσ locus contains ten V followed by two tandem copies of J-C with a V in between them. Whether this is a translocon organization is debatable. Except for in the stickleback, all IgLσ and IgLλ V have been found in the same transcriptional orientation as their corresponding J and C genes [56].

The different teleost IgH loci have undergone multiple rounds of duplication and deletion. Briefly, the bony fish IgH genes are highly diverse both in terms of the number of duplications and the types of duplications they have acquired. There are duplications of individual V_H_, D, or J segments and tandem duplications of exons encoding C_H_ domains such as the (δ2-δ3-δ4) duplications found in catfish, zebrafish, and Atlantic salmon. The IgH gene duplications in bony fish greatly contribute to diversity since duplicated genes can drift and perhaps acquire new binding functions [56].

Teleost IgH loci show great diversity amongst different groups and even individual species. For example, Salmonids such as the Atlantic salmon and rainbow trout possess “isoloci” at IgH, that are resultant of the genomic duplication (tetraploidization) experienced by the Salmonidae family. The genomic organization of the duplicated IgH loci in Atlantic salmon is distinct from that of other fish. The two IgM sub classes are both expressed (at least at the mRNA level) in some salmonids but only one isolocus is in others [16]. The loci possess eight IgT C genes 5’ of the IgM C genes, with variable numbers of Dτ and Jτ genes showing evidence of tandem duplications, with three of the Cτ genes being functional. However only one Dµ/δ-Jµ/δ-Cµ-Cδ region is seen per isolocus. Over 300 V_H_ genes exist in this salmonid, at the time a record amongst vertebrates [77,151].

In the coelacanth IgH locus, there are tandem duplications of V_H_-D units upstream of multiple duplicated JH [56]. In bony fish, alternative mRNA splicing gives rise to the IgH chain isotypes IgM and IgD as is the case in tetrapods. This is not so for the IgT isotype, which alternative rearrangement dictates in a manner akin to the αδ TCR locus [118,119]. The V region of the fugu IgH locus consists of at least 48 IgH V elements in five different families (IGHV1-IGHV5), seven IgH D genes, and six IgH J genes [152]. In the zebrafish there are 47 IgH V gene segments; 39 of these are functional, and these are divided into 13 V_H_ families based on members sharing at least 70% nucleotide identity [119]. In the stickleback (*Gasterosteusa culeatus*) the IgH gene locus is arranged in a configuration of (V_n_-D-J-Cζ-D_3_-J_4_-Cμ-Cδ)_3_-V_6_-D-J-C_ζ_, which is structurally different from any of the known teleost IgH loci. The μ genes consistently exhibit a four C encoding structure and all the ζ genes encode only three C domains (lacking the equivalent exon of the zebrafish ζ C_H_2). As in many other teleosts, the stickleback δ genes contain multiple C exons, but exist as three copies. The members of four V gene families, containing 47 segments, were interspersed in the germline [153].

In fish, membrane Igµ transcripts splice their TM exons 3’ of the Cµ3 exon, they therefore do not contain the carboxy-terminal Cµ domain (Cµ4) that is found in the SEC Igµ transcripts. The medaka membrane Igµ lacks both Cµ3 and Cµ4 domains due to the direct splicing to the TM exon to the 3’ end of the of the Cµ2 domain. VDJ-Cµ1-Cµ2-Cµ3-TM1-TM2 mRNA is seen in D. rerio, as well as an alternative VDJ-Cµ1-TM1-TM2 Igµ transcript, which encodes only one C_H_ domain [77,154].

In the catfish, the IgH locus contains 55 V, 6 D, and 12 J genes and the functional C genes for Sec and Tm forms of IgM and the Tm form of IgD. The channel catfish IgH region contains three µ/δ loci. Igτ/ζ has not been found so far. Over half of the 55 V_H_ are pseudogenes that appear non-functional. In contrast, 40 of 48 D. rerio V_H_ are functional [155]. The catfish IgH locus contains multiple Ig gene duplications and transpositions and spans ~1 mega basepair. It contains three Igδ genes (IGHD1, IGHD2, and IGHD3), and each is individually linked to either a functional Igμ gene or an Igμ pseudogene. The Igδ that encodes the IgD transmembrane form is directly 3’ of the functional Igμ gene. The other Igμ genes found in the IGH2 and IGH3 loci are pseudogenes, but the Igδ genes linked to them are intact. Furthermore, the catfish IgD secreted form is encoded by the Igδ linked to IGH3, and the membrane IgD and the (V-less) secreted IgD are always produced from the two different functional Cδ [56,77].

The Dτ-Jτ-Cτ clusters encoding IgT specific genes are generally located between the region containing theV_H_ genes and the Dµ/δ-Jµ/δ-Cµ-Cδ locus. This structure is found for example in the zebrafish, grass carp, and fugu. In this case, the configuration of IgH loci imposes the alternative production of either IgT or IgM/D rearrangements at a given locus since the recombination of V_H_ to Dµ deletes the Dτ-JτCτ regions. Since most V_H_ genes are located upstream of both DHτ and Dµ/δ, they can probably be used by IgT, IgM, and IgD [118,119].

In rainbow trout and zebrafish, the Igτ gene is located 5′ of the D and J gene segments associated with the Igμ and Igδ genes, and in these species, these Igτ genes have their own set of 5′ D and J gene segments. In both species, the Igτ genes consist of four Cτ exons and two τTM exons, and of course, this gene encodes both the secreted and transmembrane forms by differential splicing of primary RNA. While the fugu Igτ locus is organized the same, the Igτ gene contains only two Cτ exons. Igτ genes have not been found in catfish and medaka, and in these species, it may have been lost when the IgH loci were duplicated or perhaps it was never there to begin with [56,118].

In the medaka genome, five regions encoding constant domains of IgM and IgD have been identified in one large locus. Surprisingly, IgM possess only two heavy chain constant domains and it is in both secreted and membrane forms. IgD only present membrane transcripts, with Cμ1 and five Cδ exons [154]. The teleost IgHδ can vary greatly. In zebrafish V_H_ gene segments are upstream of tandem clusters of D, J, and C gene regions. While zebrafish IgM and IgZ C use the standard six exons, IgD uses a record 17 with four repeated blocks of the IgD C2-C3-C4 encoding exons [156]. In channel catfish, the IgH locus encodes two distinct δ genes that represent both the membrane-bound and secreted forms of IgD. The Igδ transmembrane form consists of VDJ-Cμ1-Cδ1-Cδ2-Cδ3-Cδ4-Cδ5-Cδ6-Cδ7-TM [118], and in rainbow trout, the full-length trout δ contains a single Ig C domain organization (μ1-δ1-δ2a-δ3a-δ4a-δ2b-δ7) with duplication of δ2-4, marked by “a” and “b” for the trout δ gene. None of the IgD clones contained the δ5 and δ6 that are typical of teleost IgD, but duplicated forms of IgD were identified [118]. Atlantic cod IgD has lost the third, fourth, fifth and sixth C encoding exons but has a duplication of the C1 and C2 domain exons. The Igδ transmembrane form consists of VDJ-Cμ1-Cδ1-Cδ2-δy-Cδ1-Cδ2-Cδ7-TM1-TM2 [113], and in the sturgeon Acipenser baeri, the lengths of sturgeon TM IgD transcripts ranged from 1.2 kb to 6.2 kb, encoding 3–19 C_H_ domains. The most prevalent Igδ membrane transcripts consist of VDJ-Cμ1-Cδ1-Cδ2-Cδ3-Cδ4-TM. However, multiple Igδ splice variants containing duplication of (Cδ2-Cδ3) also occur in sturgeons, and the longest transcripts contain eight such duplications [56,157].

The coelacanth IgH locus contains two highly distinctive IgW-encoding genes that display tandem duplications in the C domain encoding exons unlike what has been observed in other species. This singular genomic organization would preclude class switch recombination as we know it. This is the only species that does not express IgM, as no locus encoding IgM has been identified in coelacanth. The IgW1 locus contains 14 closely linked V_H_ and D gene segments and 26 diverse J_H_ gene segments upstream of the exons that encode the IgωC region. The first seven exons are unique and encode the secreted IgW. These exons are followed by four tandem duplications of (Cω3-Cω4-Cω5) and two exons encoding the TM. The adaptive immune genes sequence identity of coelacanth more resembles that in tetrapod rather than teleost [51].

The closest extant relatives of amphibians and other tetrapods are the Dipnoi (represented by the lungfish). The lungfish Ig include two relatives of IgW (given the monikers IgN and IgQ) in addition to IgM and IgW. Lungfish IgM draws from a single V. The IgH locus with corresponding IgN consists of one V domain, a DJ region, and two C domains. The other IgH locus in this organism includes a single V domain, a DJ region, and seven C domains. The recent one was phylogenetically related to cartilaginous fish IgW. It seems that IgQ is more related to sturgeon and teleost IgD and the IgW of sharks [55]. But in a recent study on two species of African lung fish, slightly different counts of IgM, IgW(D), IgN and IgQ genes were found in *P. dolloi* versus *P. annectens* with IgQ only being found in *P. annectens*. Also 17 V_H_ families in *P. annectens* and 7 in *P. dolloi* was identified. All isotypes can express IgH TM form except for IgM2 [53].

Bony fish IgL chain genes can occur in translocon organization or cluster organization; this suggests that extensive receptor editing can occur for IgL chain genes in all fish [136,137,158,159].

## 7. Fish Ig Repertoire Analysis

Fish can develop a memory response before a second exposure to an antigen. Rainbow trout respond to suboptimal doses of both T lymphocytes in an antigen-dependent and independent manner after an initial exposure to the same antigen. It is remarkable that it takes two exposures before the fish responds to the second administration of T-dependent antigens, whereas T-independent antigen requires only one exposure. While response is faster and of larger magnitude than the primary response, the number of antigen-specific B cells in the spleen is directly proportional to the frequency of B-cell specific antigen precursors. This finding suggests that the secondary response is caused by the expansion of the pool of memory B cells [9].

It was initially considered that teleost responses lacked memory as the same increase in affinity, faster kinetics and isotype switch observed in mammals was not as easily demonstrated. This was a premature conclusion, however [89]. In a rainbow trout *Streptococcus* immunization experiment, affinity increased by a factor of 10, the IgM titer increased by a factor of 100 and the diversity of antigenic proteins recognized increased by a factor of five [160]. In another study whereby trout were immunized with trinitrophenol-keyhole limpet hemocyanin, the highest affinity subpopulations emerged much later (post week 15), and had comparable titers to the intermediate affinity group [161]. Also in another study on trout, using the partition-based immunoassay, affinity maturation was independently confirmed in this teleost model [97].

Detailed repertoire analysis of IgM, IgD and IgT in rainbow trout challenged with viral hemorrhagic septicemia virus (VHSV) showed clonal expansions of IgM and IgT responses. Spleens of infected animals showed a more complex IgM repertoire than IgT, IgM using all V_H_ subgroups and dominated by a few large private and public clones. IgD and IgT repertoires in fish suggested analogous function with orthologous IgD and (functionally analogous?) IgA of mammals [162].

It is hypothesized that both IgM and IgNAR in shark are functional analogs of mammalian IgG and require T cell help. Once a high titer specific response is induced by shark immunization, it can take several years for the titer to decline to pre-immunization levels. Memory was demonstrated in sharks as reimmunization after titer fall causes titer climb with faster kinetics upon secondary response than the primary immunization [26,35].

## 8. Conclusions

In fish, not only was our IgSF-based adaptive immune system born, but B cells evolved multiple specialized isotypes of Ig and the basic mechanisms of RAG and AID mediated immunogenetic diversification used by mammals. We should continue to study the humoral response of extant fish not only to understand how the system evolved and can be applied for aquaculture, but also to see what immunobiology there might be of use to biotechnology and mammalian translational research. One particularly exciting future direction has been recently opened up by the finding of some sharks living multiple centuries [163]. The prospect of studying the repertoire dynamics of different isotypes and specific antigen responses in large vertebrates over several human lifetimes may provide a unique model to explore the boundaries of the temporal specificity and memory of adaptive immunity.

## Abbreviations

The following abbreviations are used in this manuscript:

BCR	B cell receptor
CDR	complementarity determining region
C_H_	constant heavy
C_L_	constant light
D	diversity
Fab	fragment antigen-binding
Fc	fragment crystallizable
GALT	gut associated lymphoid tissue
Ig	immunoglobulin
IgH	immunoglobulin heavy chain
IgL	immunoglobulin light chain
IgSF	immunoglobulin superfamily
J	joining
MHC	major histocompatibility complex
MYA	million years ago
N	non-template
P	palindromic
RAG	recombination activating gene
SALT	skin associated lymphoid tissue
Sec	secreted
SHM	somatic hypermutation
TCR	T cell receptor
TdT	terminal nucleotidyl transferase
Tm	transmembrane
V	variable
V_H_	variable heavy
V_L_	variable light
VLR	variable lymphocyte receptor
